# Interventions to improve oral endocrine therapy adherence in breast cancer patients

**DOI:** 10.1007/s11764-023-01513-y

**Published:** 2024-01-17

**Authors:** Sourab Ganna, Sama Rahimi, Anh Lu, Krista Laborde, Meghana Trivedi

**Affiliations:** 1 Department of Pharmaceutical Health Outcomes and Policy, University of Houston College of Pharmacy, Houston, TX 77204 USA; 2 West Penn Hospital, Pittsburg, PA 15224 USA; 3 Department of Pharmacy Practice and Translational Research, University of Houston College of Pharmacy, Health 2, 4349 Martin Luther King Blvd, Houston, TX 77204-5000 USA

**Keywords:** Breast cancer, Endocrine therapy, Medication adherence, Compliance, Text-based interventions, Technology-based interventions

## Abstract

**Purpose:**

Oral endocrine therapy (OET) is recommended in prevention and treatment of hormone receptor-positive breast cancer (HR+ BC). Despite the reduced incidence, recurrence, and mortality, OET adherence is poor in this patient population. The aim of this study was to review the latest literature to identify effective interventions to improve medication adherence in patients taking OET for prevention or treatment of HR+ BC.

**Methods:**

The Preferred Reporting Items for Systematic Reviews and Meta-Analysis (PRISMA) framework was used to perform this review. We utilized PubMed, SCOPUS, EMBASE, Cochrane, and Web of Science to acquire articles using search terms including breast cancer, adherence, persistence, and acceptability. Inclusion criteria included publication in peer-reviewed journal, primary data source, longitudinal, patients on OET such as aromatase inhibitors (AIs) or selective estrogen receptor modulators (SERMs), measuring adherence, persistence, or acceptability.

**Results:**

Out of 895 articles identified, 10 articles were included. Majority of patients had early-stage HR+ BC. Two out of two studies incorporating technological intervention, two out of three studies with text communication-based intervention, and three out of five studies with verbal communication-based intervention reported significant improvement in OET adherence and/or persistence.

**Conclusions:**

While the interventions tested so far have shown to improve OET adherence in HR+ BC patients in some studies, there is a need to design combination interventions addressing multiple barriers in this population.

**Implications for Cancer Survivors:**

This study showcases effectiveness of novel interventions to improve OET adherence and the need to further develop patient-centered strategies to benefit all patients with HR+ BC.

**Supplementary Information:**

The online version contains supplementary material available at 10.1007/s11764-023-01513-y.

## Introduction

Worldwide, 2.26 million women were newly diagnosed with breast cancer (BC) in 2020 making BC the most common type of cancer in women globally [1, 2]. It is also the second most common type of cancer in the United States (U.S.). Compared to lung and colorectal cancers, BC has a higher overall survival rate due to the availability of screening programs for early detection and effective prevention and treatment options [3]. Oral endocrine therapy (OET) is the standard mode of treatment in patients with hormone receptor-positive (HR+) BC, comprising of 70–80% of all BCs [4]. Common OETs include selective estrogen receptor modulators (SERMs) such as tamoxifen, or aromatase inhibitors (AIs) such as anastrozole, letrozole, and exemestane [5]. Five years of therapy with tamoxifen can reduce 15-year risks of BC recurrence by as much as 40% and mortality by 30% [6]. In general, AIs have demonstrated better recurrence and survival benefits than tamoxifen [7]. Ten years of adjuvant OET has been linked to superior outcomes than 5 years of therapy and is encouraged by clinicians in at least some patients [8–10]. Even in the prevention settings, OETs have indicated a consistent reduction in the risk of developing HR+ BC in high-risk women [11–14].

Despite the clinical benefit of OET, numerous studies have continuously reported less-than-ideal adherence rates (typically set at 80%) in patients in the real-world setting. In 2010, a cohort study reported that only 49% of U.S. patients were adherent to OET during the 5-year treatment period [15]. In 2012, a systematic review of 29 studies found prevalence of OET adherence ranged from 41 to 72% with discontinuation rates anywhere from 31 to 73% [16]. In 2021, a retrospective cross-sectional Surveillance, Epidemiology, and End Results (SEER) database study indicated an average 1-year adherence rate of 87% with a 5-year adherence rate dropping to 65.2% [17]. The practice of prescribing preventive therapy is also still far from ideal with utilization in 2013 only at 14.7% [18].

Non-adherence to OET is affected by many patient-, treatment-, or healthcare system–related factors [4, 19]. While there is an abundance of evidence identifying barriers to OET adherence, effective interventions to improve OET adherence are lacking. Our earlier systematic review looking at interventions to improve adherence in BC patients on OET published in 2018 found no effective intervention strategy [20]. As the number of BC patients continues to rise worldwide, there is a dire need for successful interventions to improve OET adherence. The aim of this review is to evaluate the latest studies looking at interventions to improve OET adherence, which is linked to improved clinical outcomes in HR+ BC patients.

## Methods

The structure of this systematic review was founded on the PRISMA 2020 Systematic Review Checklist [21]. The primary research question was created based on the PICO method (population, intervention, comparator, and outcomes). The population of interest included patients on OET for BC prevention or treatment. The intervention was defined as any intervention not limited to education, counseling, technology, communication, etc. The comparator was standard of care or no active intervention. The outcomes evaluated were any measure of adherence, persistence, and acceptability to OET between the intervention and comparator.

### Search strategy

The literature search and evidence extraction were performed by three researchers. The database used to identify articles was PubMed, SCOPUS, EMBASE, Cochrane, and Web of Science. Following the PICO format, the final search strategy used search terms based on the primary research question which is provided in Supplementary Table 1. To update our findings published earlier [20], the search was restricted from July 1, 2017, to July 31, 2023.

### Data collection and selection process

Once the search strategy yielded the final list of articles, a review of titles and abstracts was conducted followed by a full-text review to obtain a final compilation of articles to synthesize the resulting evidence. An electronic review tool, Rayyan.ai software [22], was used to curate evidence. A brief orientation to the electronic tool was carried out to ensure all researchers could use the electronic review tool by all those involved.

### Study inclusion and exclusion criteria

The curation of evidence at the title and abstract level along with the full-text review was carried out in accordance with the following inclusion and exclusion criteria. Inclusion criteria were (1) articles in English; (2) publication in a peer-reviewed journal; (3) utilization of primary data; (4) prospective and longitudinal study design; (5) inclusion of patients prescribed or initiated on OET including SERMs such as tamoxifen, raloxifene, or AIs such as anastrozole, letrozole, or exemestane for BC prevention or treatment; (6) the outcome measures including adherence, persistence, or acceptability; and (7) active interventions. Exclusion criteria were (1) non-randomized clinical trials, (2) studies without a comparator, and (3) publication types including reviews, protocols, editorials, or commentaries.

### Quality and risk of bias assessment of selected studies

Qualitative analysis of the study design was performed using the modified Downs and Black 27-item methodological scale with a maximum possible score of 28 points for randomized and non-randomized health interventions as done in other studies [23–25]. Each article was scored based on “poor” (≤ 14) or “appropriate” (15–28 points). Two investigators ranked the studies, and those scoring below 15, or as “poor,” were excluded from the final analysis.

### Outcome measures

Characteristics of included studies and patients were summarized descriptively including sample size, age, race/ethnicity, OET medications prescribed, tumor stage of study subjects, and medication/surgical history. Data collected from studies included types of interventions, specific interventions, and study results. Primary outcome measures included adherence, persistence, and acceptability to OET. Adherence was evaluated as the degree to which patients adhere to their recommended OET treatment plans. Various assessment methods included self-reported measures, electronic monitoring devices, pill counts, and pharmacy refill records. Persistence was measured as the duration for which patients continuously adhered to their OET treatment plans over time. Acceptability was measured as patients’ willingness or openness to adopt and engage with the intervention. Secondary outcomes included any other measure related to OET adherence, persistence, or acceptability. Each of the above outcome measures was grouped and reported by technological interventions, text communications, or verbal communications.

## Results

### Study selection

**Fig. 1:**
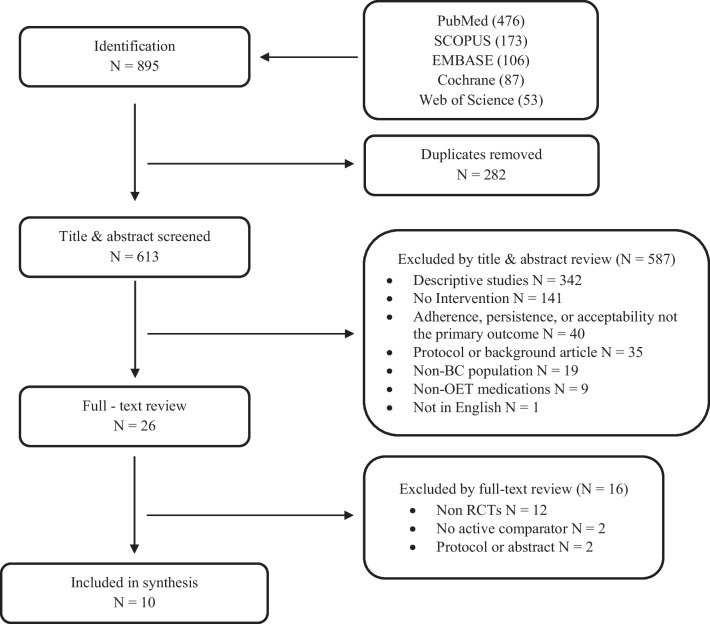
PRISMA flow diagram for studies looking at interventions to improve adherence in patients on OET for BC. BC, breast cancer; OET, oral endocrine therapy; PRISMA, Preferred Reporting Items for Systematic Reviews and Meta-analyses

Figure 1 shows the systematic process of retrieving, screening, and the final selection of the articles based on the search strategy. Of the original 895 articles collected, 282 articles were removed as being duplicates resulting in an overall 613 articles, which underwent title and abstract screening. A total of 587 articles did not meet the inclusion criteria and were excluded. Of the 26 full-text articles reviewed, 16 were excluded because they either were non-randomized controlled trials (RCTs) (*n* = 12), had no comparator (*n* = 2), or were an abstract or protocol for a potential study (*n* = 1). Finally, 10 articles were included in the final synthesis of this review, all of which also met the appropriate Downs and Black scoring criteria.

### Risk of bias in studies

Regarding the Downs and Black’s scoring for potential risk of bias, the studies varied widely in methodological quality; all the results meeting inclusion and exclusion criteria had an overall score ranging from 18 to 25 with a median score of 21. The median score for reporting bias was 9 with a range from 7 to 10. External validity had a median of 3 with no range. External validity had a median of 4 with a range of 3 to 5. Selection bias had a median of 4 with a range from 3 to 6. Power was only captured in four studies. All 10 studies met the appropriate Downs and Black scoring criteria (Table 1).

**Table 1:** Downs and Black scores for all included studies assessing the methodological quality across a variety of categories

First author (year) [ref]	Reporting bias	External validity	External validity bias	Selection bias	Power	Total score
	(0–10)	(0–3)	(0–7)	(0–6)	(0–1)	
Park (2022) [26]	10	3	4	4	0	21
Graetz (2018) [27]	7	3	4	4	0	18
Tan (2020) [28]	9	3	4	4	0	20
Singleton (2022) [29]	9	3	5	4	0	21
Hershman (2020) [30]	10	3	5	6	1	25
Arch (2022) [31]	9	3	4	4	1	21
Ream (2021) [32]	9	3	5	5	0	22
Getachew (2022) [33]	8	3	3	3	1	18
Jacobs (2022) [34]	10	3	4	4	1	22
Riis (2020) [35]	10	3	4	5	0	22

### Study characteristics

The study and patient characteristics are summarized in Table 2. Of the 10 RCTs included, six studies included patients on both AIs and SERMs, one study included patients on only SERMs, and three studies included patients on only AIs. Five studies were single-centered [27, 29, 32, 34, 35], while five were multi-centered studies [28, 30, 31, 33, 34]. Five studies were conducted in the U.S. [27, 30–32, 34], and five were conducted internationally: Australia [29], Denmark [35], Ethiopia [33], Singapore [28], and South Korea [26]. Patient enrollment ranged from the smallest study enrolling 44 subjects [27] to the largest study enrolling 702 subjects [30]. The duration of intervention ranged from 4 weeks to 8 years among the studies measuring adherence, persistence, and acceptability. Most patients included in these studies were Non-Hispanic White and were diagnosed with stage I or II BC. As summarized in Table 3, two studies used technology-based interventions [26, 27], three used text-based communication [28–30], and five used verbal communication [31–35]. While all studies reported adherence as one of the outcomes, one study reported persistence [33], and only four studies reported acceptability of the intervention(s) [28, 31, 34, 35].

**Table 2:** Study and patient characteristics

Characteristic	Park (2022) [26]	Graetz (2018) [27]	Tan (2020) [28]	Singleton (2022) [29]	Hershman (2020) [30]	Arch (2022) [31]	Ream (2021) [32]	Getachew (2022) [33]	Jacobs (2022) [34]	Riis (2020) [35]
Sample, *n*	61	44	244	160	702	88	59	162	100	134
Age, mean	53.3	59.9	61	54.8	60.9	58.3	54.2	C: 38.5, I: 41.8	56.1	C: 64.2, I: 64.4
Race/ethnicity, %										
White/non-Hispanic	N/A	75	0	30	89	92	47	N/A	91	N/A
White/Hispanic	N/A	2	0	N/A	0	3	40	N/A	3	N/A
Black/African American	N/A	23	0	N/A	7	0	9	N/A	1	N/A
Native American/Alaskan Native	N/A	N/A	0	N/A	0	1	N/A	N/A	N/A	N/A
Asian	N/A	N/A	100	50	2	N/A	N/A	N/A	4	N/A
Other	N/A	N/A	0	20	2	4	4	N/A	1	N/A
OET medication(s), %										
Tamoxifen	56	N/A	N/A	100	N/A	40	100	C: 18, I: 5	40	C: 94, I: 97
Raloxifene	N/A	N/A	N/A		N/A	N/A		N/A	N/A	
Anastrozole	30	100	100		72	60		N/A	60	
Letrozole	14				24			N/A		
Exemestane	N/A				4			N/A		
Duration (intervention)	4 wks	8 wks	1 yr	6 mos	3 yrs	6 mos	8 yrs	1 yr	24 wks	2 yrs
Tumor stage, %										
Stage 0	61	21	0	N/A	N/A	5	9	N/A	7	N/A
Stage I		59	40	N/A	63	72	56	C: 7, I: 7	73	N/A
Stage II	26	11	38	N/A	37	18	27	C: 47, I: 23	15	N/A
Stage III	13	9	20	N/A		5	8	C: 41, I: 65	5	N/A
Stage IV	N/A	N/A	N/A	N/A	N/A	N/A	N/A	N/A	N/A	N/A
Unknown	N/A	N/A	2	N/A	N/A	N/A	N/A	C: 5, I: 5	N/A	N/A
Medication/surgical history, %										
Chemotherapy	N/A	N/A	N/A	63	44	34	N/A	C: 21, I: 17	8	C: 48, I: 39
Mastectomy	21	N/A	N/A	99	N/A	100	N/A	N/A	23	C: 22, I: 23
Lumpectomy	79	N/A	N/A		N/A	N/A	N/A	N/A		C: 78, I: 77
Radiation	N/A	N/A	N/A	89	66	68	N/A	C, 7; I, 8	48	C: 90, I: 89

*C* = control, *I* = intervention, *mos* = months, *N/A* = not applicable, *OET* = oral endocrine therapy, *wks* = weeks, *yrs* = years

**Table 3:** Study results of published articles addressing various interventions implemented aimed to improve OET adherence in BC patients

Study, author (year) (sample size) [ref]	Intervention	Primary outcomes result(s)	Other outcomes result(s)
Technological interventions			
Park (2022) (*N*=61) [26]	I: Smart pill bottle with Pillsy mobile application, C: No intervention	Adherence at 28 days (Pillsy mobile app)*: I: 97%, C: 88%	Self-efficacy: *F* = 9.07
Graetz (2018) (*N*=44) [27]	I: Mobile application + reminders, C: Mobile application	Adherence at 8 wks (MMAS-4)*: I: 100%, C: 72%	App usage*: I: 38%, C: 74%; Symptom burden: DID = 7.6
Text communications			
Tan (2020) (*N*=244) [28]	I: EMPOWER-SMS, C: No intervention	Adherence at 1 yr (SMAQ)*: OR 2.35 95% CI [1.01–5.49] Acceptability: 79–99%	
Singleton (*N*=160) (2022) [29]	I: Text message reminders providing education, C: No intervention	AF at 6 mos (SR)*: I: 7%, C: 17%, aRR 0.13 95% CI [0.02–0.91]	Self-efficacy (SEMCDS)*: I: 7.1%, C: 7.4%; QoL: C: I: 69%, 70.4%
Hershman (2020) (*N*=702) [30]	I: Twice weekly text messages, C: No intervention	AF at 3 yrs (BA): I: 82%, C: 87%	Time to AF at 3 yrs: HR: 1.16 [95% CI, 0.69–1.98]
Verbal communication			
Arch (2022) (*N*=88) [31]	I: REACH + education, C: Education alone	Adherence at 6 mos (Wisepill)*: C: 95%, I: 96%; attitudes at 3 months: I: 0.97, C: 0.99	Feasibility: 93.02% chose to view the adherence graph at least one
Ream (2021) (*N*=59) [32]	I1: Relaxation training, I2: CBT, C: Health education	Adherence at 8 yrs (ETMUQ)*: B(SE) = 0.25 (0.14)	
Getachew (2022) (*N*=44) [33]	I: Nurse counseling, phone call reminders, and medication monitoring, C: No intervention	Adherence at 1 yr (SMAQ)*: I: 70%, C: 45%; Persistence*: I: 91%, C: 78%	
Jacobs (2022) (*N*=100) [34]	I: STRIDE, C: No intervention	Adherence at 12 wks (MARS-5): I: 24, C: 24; satisfaction (CTSQ): I: 66.5C: 64	Symptom distress (BCPT-SCL)*: I: 6.0, C: 5.3
Riis (2020) (*N*=134) [35]	I: Patient-initiated follow-up care, C: No intervention	Adherence at 2 yrs (EMR): I: 98%, C: 96%; satisfaction (PEQ): I: 98%, C: 95%	QoL: No difference

*AF * = adherence failure, *aRR* = adjusted relative risk, *BA* = biological urine assay measuring drug and metabolite levels in urine, *BC* = breast cancer, *BCPT-SCL* = Breast Cancer Prevention Trial Symptom Checklist, *C* = control, *CBT* = cognitive behavioral therapy, *CTSQ* = Cancer Therapy Satisfaction Questionnaire, *DID* = difference-in-difference, *EMPOWER-SMS* = lifestyle-focused text message intervention, *EMR* = electronic medical records, *ETMUQ* = endocrine therapy medication usage, *I* = intervention, *MARS-5* = Medication Adherence Report Scale, *MMAS-4* = Morisky Medication Adherence Scale, *mos* = months, *N/A* = not applicable, *OET* = oral endocrine therapy, *PEQ* = Patient Experience Questionnaire, *QoL* = quality of life, *REACH* = Resources and Education for Adherence to Cancer Hormonal therapy, *SEMCDS* = Self-efficacy for Managing Chronic Disease Scale, *SMAQ* = Simplified Medication Adherence Questionnaire, *SMS* = short messaging service, *STRIDE* = Symptom-Targeted Randomized Intervention for Distress and Adherence to Adjuvant Endocrine Therapy, *SR* = self-report, *wks* = weeks, *yrs* = years

### Interventions

#### Technological interventions

In the two studies utilizing technological interventions, one included smart pill bottles with the Pillsy mobile application [26] while the other utilized provision of information such as weekly adherence reminders through a mobile application [27]. In both studies, technological interventions significantly improved adherence in comparison to no intervention in the control group (Table 3). However, self-efficacy was not significantly higher with the Smart pill bottle used for 4 weeks in 61 patients. Mobile app weekly reminders for 8 weeks had significantly higher usage among patients in the intervention compared to the control group without significant improvement in symptom burden in 44 patients.

#### Text communications

In the studies utilizing text communication, the studies included personalized or standardized unidirectional text reminders to the patient’s designated mobile device with information regarding adherence sent by a nurse or through an automated system at scheduled intervals [28–30]. Two out of three studies reported significant improvement (Table 3). SMS reminder texts with notifications to take OET significantly improved adherence at 1 year in 244 patients in one study [28] with the majority of patients also thought that the intervention was easy to understand (99%), useful (79%), and provided enough information (97%). Additionally, 86% recommended it as a part of routine care [28]. The use of text reminders providing health education also significantly reduced the probability of missing more than one dose of OET as scheduled after 6 months in 160 patients in another study [29]. Self-efficacy and quality of life were largely indifferent between control and intervention arms. However, in a larger study with 702 patients, text messages sent twice a week focusing on barriers to adherence, cues to action, efficacy, and reminders were not able to significantly reduce adherence failure rates at 3 years [30].

#### Verbal communications

Studies employing verbal communication as the intervention included any in-person discussion over various topics regarding the patient medication regimen between a healthcare provider and patient in various settings of which three studies reported significant improvement (Table 3) [31–35]. The Resources and Education for Adherence to Cancer Hormonal therapy (REACH) intervention combined with education significantly improved adherence compared to education alone after 1 month of follow-up in 88 patients [31]. While the REACH + education cohort continued to demonstrate better adherence until month 4 of follow-up, the difference was not statistically significant at the later time point. In addition, lower negative attitudes were observed in the REACH + education arm for the first 3 months. In another study, relaxation training significantly reduced the likelihood of forgetfulness and intentional nonadherence compared to cognitive behavioral therapy (CBT) or health education in 59 patients [32]. Nurse-led counseling sessions in addition with phone call reminders and medication monitoring significantly improved adherence and persistence in 87 patients in another study [33]. The symptom-targeted randomized intervention for distress and adherence to adjuvant endocrine therapy (STRIDE) intervention using 6 weekly video conferencing sessions with 2 individual calls significantly improved symptom distress without any effect on OET adherence or satisfaction among 100 patients [34]. However, patient-initiated follow-up care was also ineffective in improving adherence, satisfaction, or QoL in 134 patients [35].

## Discussion

This review highlights current studies that have aimed to evaluate various types of interventions to improve OET adherence, persistence, and acceptability in HR+ BC patients. Our current review found seven of the 10 studies provided a significant improvement in the primary outcome of adherence, while the remaining 3 either indicated a trend towards improvement or improvement in other outcomes. Both studies evaluating technological interventions involving mobile application-based reminders significantly improved OET adherence. Two studies with text communication-based interventions showed significant improvement in OET adherence at 6 or 12 months, whereas one study measuring adherence at 3 years did not. Three of five verbal communication-based intervention significantly improved OET adherence or persistence.

In our previous review in 2018, we reviewed 5 studies, none of which reported a significant improvement in OET adherence with educational material as an intervention in HR+ BC patients [20]. Since then, recent literature has considered advances in technology and incorporated other methodologies that also include bidirectional communication with health care providers to improve adherence barriers. Bidirectional social support has reported higher levels of acceptability and educational, physical, and emotional benefits in a qualitative semi-structured interview of BC patients on OET for 12 months across three states in the USA [36]. In accordance, future research could investigate exploring interventions that combine the aspects of technology, text, or verbal interventions to address multi-factorial barriers. For example, technology and text messaging can help address forgetfulness, whereas both text and verbal communications can improve patient-clinician relationship and provide education on importance of OET. Frequent check-ins may also be required to ensure the effectiveness of these intervention over time. The customized intervention should also have the feature of flexibility to address the changing needs of patients. Hence, a structured plan that incorporates various methods, check-ins, and adaptability to modify the interventions is necessary to improve OET adherence, persistence, and acceptance in HR+ BC patients.

While most studies have focused on improving adherence to OET in treatment of HR+ BC patients, an emphasis on OET adherence in the prevention setting for patients who are at higher risk of developing BC is lacking. Literature has shown preventative measures are able to reduce the burden on the patient and healthcare system clinically and economically when compared between patient cases diagnosed at early and advanced stages [37]. However, utilization of OET as prevention strategy is largely underutilized to its full capacity for a variety of reasons including, but not limited to, a lack of physicians’ and patients’ knowledge of available cancer risk evaluation tools or prevention measures, and underestimation of the benefits complemented with the overestimation of risk of taking the medications [37, 38]. Even among the patients taking OET for BC prevention, the adherence is poor and need to be improved to help reduce the overall incidence and severity of BC in the chronic setting [37, 39].

Potential limitations of the studies included here are as follows: first, most studies had a relatively short follow-up period of 1 year or less. OET is typically given to patients anywhere from 5 to 10 years [9]. While longer follow-up periods should provide more information regarding the efficacy of the intervention, it is also likely after time a single intervention alone is no longer efficacious. For example, one study in our review with a follow-up of 3 years did not significantly improve OET adherence [30]. Potentially multiple interventions are needed to maintain adherence superiority at longer follow-up periods. In addition, apart from one study in this review measuring true adherence with devices such as MEMS Caps, all studies measured adherence indirectly. This can be potentially problematic given that adherence may be over-estimated with the use of patient-reported adherence, rating scales and surveys, or pill counts by as much as 17% [40]. Future studies should look to evaluate past and future interventions using true adherence measured by devices at the time of administration to truly capture the effect of the intervention. Lastly, none of the studies reported any information on the fidelity of the interventions, which is a critical aspect when considering if the outcome of an intervention is due to the clinical aspect of the lack of proper implementation and delivery of the intervention.

An assessment of why an intervention succeeds or fails would better enable clinicians in deciding whether to utilize it in clinical practice. Using theoretical models such as the Anderson Behavior Model (ABM) when developing adherence interventions and methods to measure adherence would increase the overall validity of the studies and help qualitatively assess the potential pitfalls of any study that did not indicate an improvement. ABM explains how an overall outcome is directly a result of patient health behaviors which consists of personal health practices and use the of health services [41]. A start would be using modern technology such as pill tracking smart bottles linked to mobile services providing information to both providers and patients [42] or by combining various complementary interventions together capitalizing on the strengths by resolving any weaknesses of the respective interventions in the structure and process portions to achieve the best outcomes of the patient [43]. Few studies go beyond adherence, persistence, or acceptability to capture personal health practices. Measures that could provide additional vital information include feasibility or utilization of the health interventions. However, further research is still needed to provide high-quality, effective, and cost-efficient interventions.

## Conclusion

In conclusion, this systematic review showcases the scope of the current literature’s effectiveness of technological, text, and verbal communications in improving OET adherence in HR+ BC patients. While these interventions have shown effectiveness in a small number of patients followed for a short period of time, more work is needed before they can be incorporated in routine practice. Future research should design and test customizable interventions that may incorporate some of the interventions discussed here, standardize adherence measurement, assess acceptability and fidelity outcomes, conduct more RCTs with larger sample sizes, and explore long-term effects of the interventions. In addition, an effort should be made to constantly improve the interventions by teams of healthcare professionals, insurance companies, hospital representatives, and private sector to design, test, and implement novel strategies seamlessly for all involved parties. By addressing these gaps, we can advance the field of OET adherence interventions and improve outcomes in HR+ BC patients.

## Supplementary information

ESM 1 (DOCX 15 kb)
