# Supplementary material for: Interventions to improve oral endocrine therapy adherence in breast cancer patients
Source: J Cancer Surviv. 2024 Jan 17;19(3):930–9. doi: 10.1007/s11764-023-01513-y (PMC12081578; doi:10.1007/s11764-023-01513-y)
Supplement: Supplementary file 1 — (DOCX 15 kb) [file 11764_2023_1513_MOESM1_ESM.docx]

**Supplementary Table 1: Search strategy.**

| **Database** | **Search strategy** |
| --- | --- |
| PubMed | (breast cancer [MeSH Terms:noexp] OR breast neoplasms [MeSH Terms:noexp] OR breast neoplasms [All Fields] OR breast cancer [All Fields]) AND (antineoplastic agents, hormonal [MeSH Terms:noexp] OR antineoplastic agents, hormonal [All Fields] OR aromatase inhibitors [MeSH Terms:noexp] OR aromatase inhibitors [All Fields] OR endocrine therapy [All Fields]) AND (patient compliance [MeSH Terms:noexp] OR patient compliance [All Fields] OR medication adherence [MeSH Terms:noexp] OR medication adherence [All Fields] OR treatment adherence [All Fields] OR acceptability [All Fields]) AND (intervention [All Fields] OR educational instruction [All Fields] OR electronic intervention [All Fields] OR behavioral therapy [All Fields] OR motivational intervention [All Fields] OR reminder systems [All Fields] OR telehealth [All Fields] OR telemedicine [All Fields] OR counseling [All Fields]) 2017:2023[dp] |
| SCOPUS | (TITLE-ABS-KEY(“breast cancer”) OR TITLE-ABS-KEY(“breast neoplasms”) OR TITLE-ABS-KEY(“breast neoplasms”) OR TITLE-ABS-KEY(“breast cancer”)) AND (TITLE-ABS-KEY(“antineoplastic agents, hormonal”) OR TITLE-ABS-KEY(“antineoplastic agents, hormonal”) OR TITLE-ABS-KEY(“aromatase inhibitors”) OR TITLE-ABS-KEY(“aromatase inhibitors”) OR TITLE-ABS-KEY(“endocrine therapy”)) AND (TITLE-ABS-KEY(“patient compliance”) OR TITLE-ABS-KEY(“patient compliance”) OR TITLE-ABS-KEY(“medication adherence”) OR TITLE-ABS-KEY(“medication adherence”) OR TITLE-ABS-KEY(“treatment adherence”) OR TITLE-ABS-KEY(“acceptability”)) AND (TITLE-ABS-KEY(“intervention”) OR TITLE-ABS-KEY(“educational instruction”) OR TITLE-ABS-KEY(“electronic intervention”) OR TITLE-ABS-KEY(“behavioral therapy”) OR TITLE-ABS-KEY(“motivational intervention”) OR TITLE-ABS-KEY(“reminder systems”) OR TITLE-ABS-KEY(“telehealth”) OR TITLE-ABS-KEY(“telemedicine”) OR TITLE-ABS-KEY(“counseling”)) |
| Ovid (EMBASE) | (“breast cancer”.mp. OR “breast neoplasms”.mp. OR “breast neoplasms”.mp. OR “breast cancer”.mp.) AND (“antineoplastic agents, hormonal”.mp. OR “antineoplastic agents, hormonal”.mp. OR “aromatase inhibitors”.mp. OR “aromatase inhibitors”.mp. OR “endocrine therapy”.mp.) AND (“patient compliance”.mp. OR “patient compliance”.mp. OR “medication adherence”.mp. OR “medication adherence”.mp. OR “treatment adherence”.mp. OR “acceptability”.mp.) AND (“intervention”.mp. OR “educational instruction”.mp. OR “electronic intervention”.mp. OR “behavioral therapy”.mp. OR “motivational intervention”.mp. OR “reminder systems”.mp. OR “telehealth”.mp. OR “telemedicine”.mp. OR “counseling”.mp.) |
| Cochrane | (“breast cancer” OR “breast neoplasms” OR “breast neoplasms” OR “breast cancer”) AND (“antineoplastic agents, hormonal” OR “antineoplastic agents, hormonal” OR “aromatase inhibitors” OR “aromatase inhibitors” OR “endocrine therapy”) AND (“patient compliance” OR “patient compliance” OR “medication adherence” OR “medication adherence” OR “treatment adherence” OR “acceptability”) AND (“intervention” OR “educational instruction” OR “electronic intervention” OR “behavioral therapy” OR “motivational intervention” OR “reminder systems” OR “telehealth” OR “telemedicine” OR “counseling”) |
| Web of Science | ALL=((“breast cancer” OR “breast neoplasms” OR “breast neoplasms” OR “breast cancer”) AND (“antineoplastic agents, hormonal” OR “antineoplastic agents, hormonal” OR “aromatase inhibitors” OR “aromatase inhibitors” OR “endocrine therapy”) AND (“patient compliance” OR “patient compliance” OR “medication adherence” OR “medication adherence” OR “treatment adherence” OR “acceptability”) AND (“intervention” OR “educational instruction” OR “electronic intervention” OR “behavioral therapy” OR “motivational intervention” OR “reminder systems” OR “telehealth” OR “telemedicine” OR “counseling”)) |
